# Supplementary material for: Association between chiropractic spinal manipulation and cauda equina syndrome in adults with low back pain: Retrospective cohort study of US academic health centers
Source: PLoS One. 2024 Mar 11;19(3):e0299159. doi: 10.1371/journal.pone.0299159 (PMC10927125; doi:10.1371/journal.pone.0299159)
Supplement: S1 Fig — (DOCX) [file pone.0299159.s001.docx]

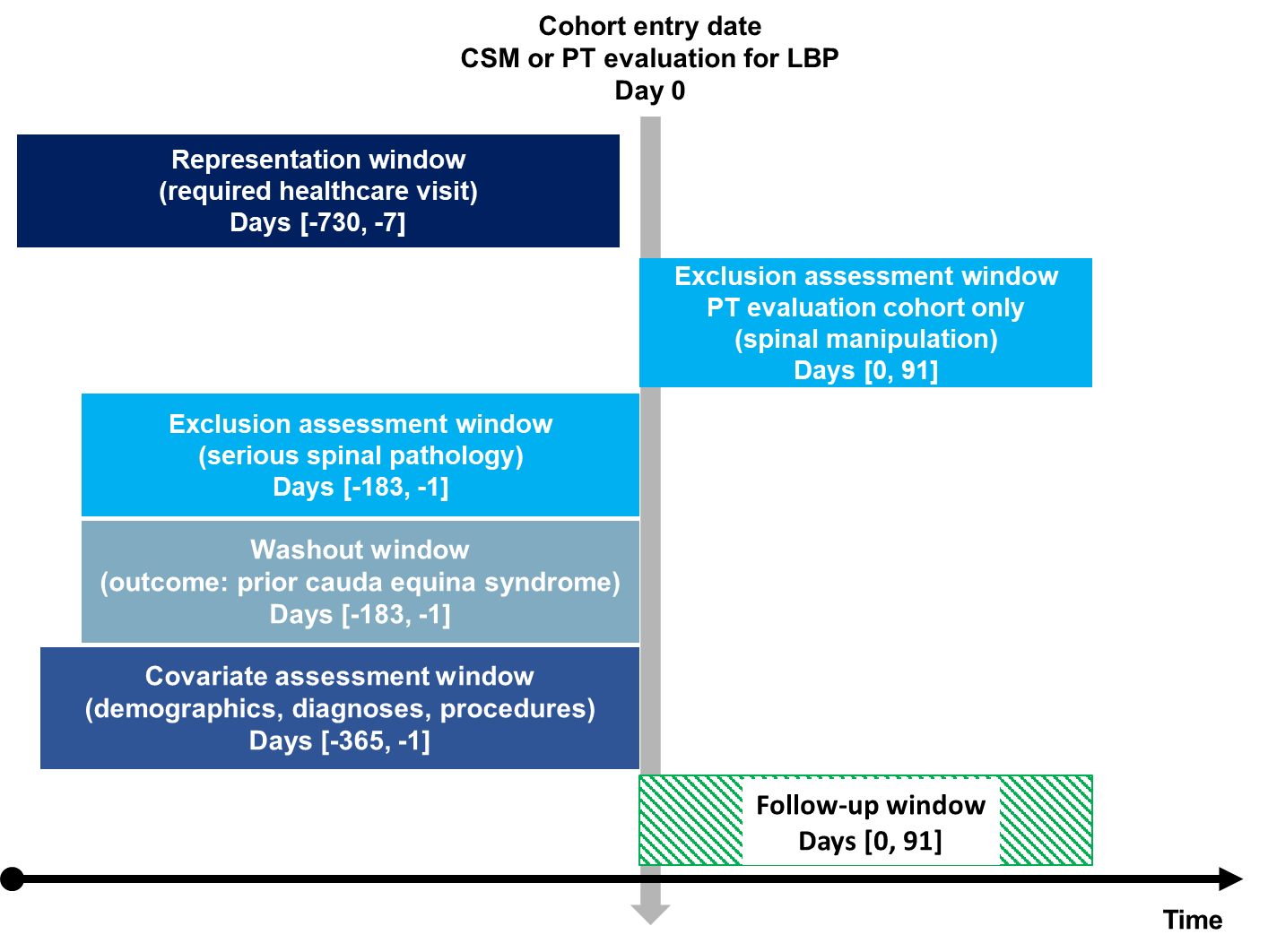


S1 Figure: Study design. The vertical arrow represents the index date of the first co-occurrence of a diagnosis of low back pain with either chiropractic spinal manipulation or physical therapy evaluation. Windows to the left of this arrow represent time periods preceding the index date over a span of days [#,#]. The window to the right of the arrow indicates events after the index date. The “∞” indicates that the time window extends as far retrospectively as data are available for each patient. Abbreviations: Chiropractic spinal manipulation (CSM), low back pain (LBP), physical therapy (PT). Image adapted by Robert J. Trager using a Creative Commons template [1].

**Reference:**

1. Schneeweiss S, A.  Rassen J, S.  Brown J, J.  Rothman K, Happe L, Arlett P, et al. Graphical Depiction of Longitudinal Study Designs in Health Care Databases. Ann Intern Med. 2019;170:398–406.
