# Supplementary material for: Association between chiropractic spinal manipulation and cauda equina syndrome in adults with low back pain: Retrospective cohort study of US academic health centers
Source: PLoS One. 2024 Mar 11;19(3):e0299159. doi: 10.1371/journal.pone.0299159 (PMC10927125; doi:10.1371/journal.pone.0299159)
Supplement: S1 Table — (DOCX) [file pone.0299159.s002.docx]

S1 Table: Inclusion codes for both cohorts

| **Diagnosis codes*** | **Definition** |
| --- | --- |
| M54.5 | Low back pain |
| M47.816 | Spondylosis without myelopathy or radiculopathy, lumbar region |
| M47.817 | Spondylosis without myelopathy or radiculopathy, lumbosacral region |
| M48.06 | Spinal stenosis, lumbar region |
| M51.26 | Other intervertebral disc displacement, lumbar region |
| M51.27 | Other intervertebral disc displacement, lumbosacral region |
| M51.36 | Other intervertebral disc degeneration, lumbar region |
| M51.37 | Other intervertebral disc degeneration, lumbosacral region |
| M54.16 | Radiculopathy, lumbar region |
| M54.17 | Radiculopathy, lumbosacral region |
| M54.18 | Radiculopathy, sacral and sacrococcygeal region |
| M54.3 | Sciatica |
| M54.4 | Lumbago with sciatica |
| *International Classification of Diseases, 10^th^ Edition (ICD-10) | |
