# Supplementary material for: Association between chiropractic spinal manipulation and cauda equina syndrome in adults with low back pain: Retrospective cohort study of US academic health centers
Source: PLoS One. 2024 Mar 11;19(3):e0299159. doi: 10.1371/journal.pone.0299159 (PMC10927125; doi:10.1371/journal.pone.0299159)
Supplement: Attachment — Submitted filename: reviewers report.docx [file pone.0299159.s005.docx]

Dear authors, I am very happy to be able to write positively. It is evident that you have put a great deal of effort into this project and I want to praise your efforts, Fortunately, the actual contribution from your study is clear and, the manuscript as currently written suggests that it might be suitable for sharing information about this topic, but the manuscript that you reported, needs few minor edits.

**General comment**: Authors must consider using line numbers in manuscripts.

**Abstract**

Line: ‘following physical therapy (PT) evaluation’: It can be better written as ‘intervention’, instead of ‘evaluation’.

‘Patients were divided into two cohorts: (1) CSM or (2) PT evaluation without spinal Manipulation’: Did the patients underwent one time evaluation (assessment) or were they on specific physiotherapeutic interventions for any specific time (such as a week, or two)? If they were on intervention(s), the word ‘evaluation’ should be replaced with the word ‘intervention’.

‘Propensity score matching controlled for covariates associated with CES (e.g., body mass index).’: correct the syntax of the sentence.

Lines ‘(mean age 51 years [SD=17]) remained after propensity matching. CES incidence was 0.07% (95% CI: 0.05-0.09%) in the CSM cohort compared to 0.11% (95% CI: 0.09- 0.14%) in the PT evaluation cohort, yielding an RR (95% CI) of 0.60 (0.42-0.86; P=.0052). Both cohorts showed a higher rate of CES during the first two weeks of follow-up.’: Elaborate the abbreviations at first notice.

**Introduction**: No comments

**Materials and Methods**

Rephrase the line ‘We included patients starting 20 years ‘prior to the query date (July 30, 2023) to maximize sample size’ for a clearer understanding.

**Results**

Table 1: Add n (%) for males and females to let the readers understand that the values are in terms of n and its corresponding percentage. Also, add SI units for BMI (kg/m^2^) and other such variables in the tables.

The level of significance (*p)* should be written in small italicized letters.

**Discussion** No comments

**Limitations & Conclusions:** No comments
